# Venous Manometry as an Adjunct for Diagnosis and Multimodal Management of Intracranial Hypertension due to Meningioma Compressing Sigmoid Sinus

**DOI:** 10.7759/cureus.4953

**Published:** 2019-06-20

**Authors:** Cletus Cheyuo, Charles L Rosen, Ansaar Rai, Christopher P Cifarelli, Rabia Qaiser

**Affiliations:** 1 Neurosurgery, West Virginia University, Morgantown, USA; 2 Neurosurgery, Central Illinois Neuro Health Sciences, Bloomington, USA; 3 Radiology, West Virginia University, Morgantown, USA; 4 Neurosurgery, Baylor Scott and White Health, Temple, USA

**Keywords:** intracranial hypertension, sigmoid sinus meningioma, venous manometry, stereotactic radiosurgey

## Abstract

Intracranial venous hypertension is a rare presentation of meningiomas in the transverse-sigmoid sinus region. We describe a case of a young patient presenting with intracranial hypertension due to a meningioma causing compression of the dominant sigmoid sinus. We were able to document the cerebral venous pressure gradient across the lesion confirming our hypothesis that compression of the sigmoid sinus from the meningioma was the cause of intracranial hypertension.

The patient is a 17-year-old male who presented with intracranial hypertension due to meningioma at the right dominant sigmoid sinus, which was treated by a Simpson grade IV surgical resection followed by stereotactic radiosurgery. Following treatment, his papilledema resolved and he remains symptom-free at 18 months.

In conclusion, venous manometry is a useful adjunct to diagnose intracranial hypertension in non-idiopathic causes of intracranial hypertension. A multimodal management approach of intracranial hypertension due to outflow obstruction from the dominant sinus led to an excellent recovery on follow up.

## Introduction

Meningiomas are benign neoplasms that arise from arachnoid cap cells. The arachnoid cap cells protrude into the venous sinuses and are most abundant around the superior sagittal sinus. The posterior fossa is the site for 7-10% meningiomas, of which 10% involve the transverse or sigmoid sinus [1]. Meningiomas are commonly asymptomatic but can present with symptoms of mass effect such as headache as well as symptoms peculiar to location. Invasion of the venous sinuses by a meningioma can cause obstruction of the venous drainage leading to increases in the intracranial pressure, and venous infarctions [2].

A rare syndrome of intracranial hypertension has been described in a few meningiomas with venous sinus invasion [2-3]. Intracranial hypertension is described as intracranial pressure greater than 20 cm H2O, which manifests clinically as headaches, nausea and vomiting as well as visual symptoms such as diplopia and vision loss over a long period of time. Diagnosis is based on clinical presentation, ophthalmological evaluation for papilledema and radiological demonstration of meningioma obstructing the sinus. Venous manometry has been used previously for diagnosing idiopathic intracranial hypertension causing headaches [4]. However, to the best of our knowledge this is the first time it has been utilized for measuring the cerebral venous pressure gradient across a lesion. We describe the case of a 17-year-old male who presented with intracranial hypertension due to a meningioma at the right dominant sigmoid sinus, which was treated by surgical debulking followed by stereotactic radiosurgery.

## Case presentation

**Figure 1:**
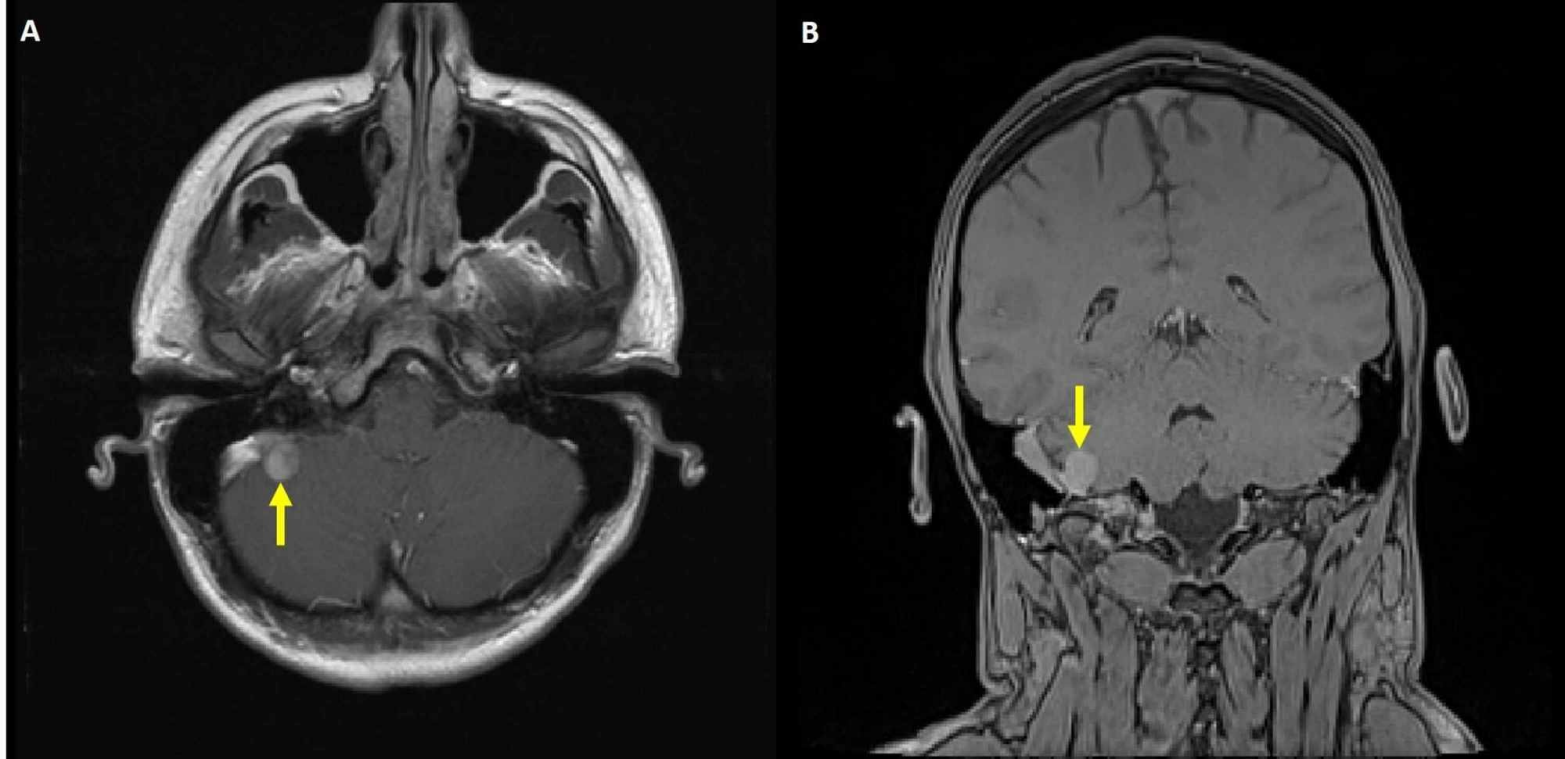
Preoperative magnetic resonance imaging (MRI) brain with gadolinium contrast (A) Axial and (B) coronal views showing an enhancing 1.5 cm extra-axial mass (solid yellow arrows) causing stenosis of the right sigmoid sinus.

The patient is a 17-year-old male who presented with 3-month history of headache, worse in the morning and associated with blurry vision, nausea and vomiting. He initially presented to an optometrist at an outside hospital, who detected bilateral papilledema and referred him for further workup. On clinical examination, he was neurologically intact. Magnetic resonance imaging (MRI) of the brain, with gadolinium contrast, showed an enhancing 1.5 cm extra-axial mass causing stenosis of the dominant right sigmoid sinus (Figure 1). 

**Figure 2:**
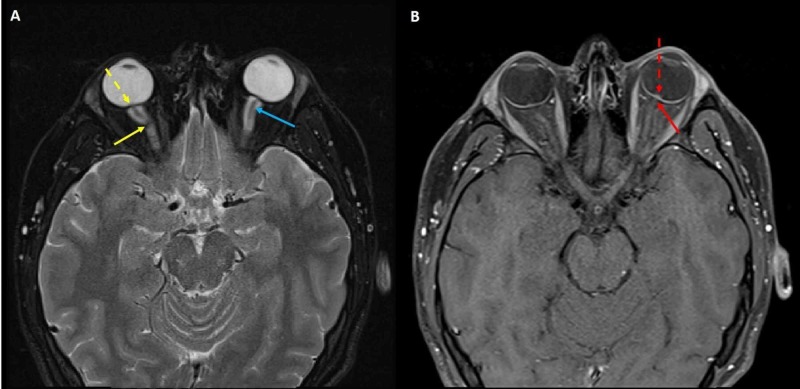
Magnetic resonance imaging (MRI) of the orbits with gadolinium contrast demonstrating papilledema (A) Axial T2W showing flattening of the posterior sclera (dashed yellow arrow), distension of the peri-optic subarachnoid space (solid blue arrow) and vertical tortuosity of the orbital optic nerve (solid yellow arrow) and (B) Axial T1W with contrast showing enhancement of the prelaminar optic nerve (solid red arrow) and intraocular protrusion of the prelaminar optic nerve (dashed red arrow).

MRI of the orbits, with gadolinium contrast, showed the classic features of papilledema [5]; including flattening of the posterior sclera, enhancement of the prelaminar optic nerve, distension of the peri-optic subarachnoid space, intraocular protrusion of the prelaminar optic nerve and vertical tortuosity of the orbital optic nerve (Figure 2).

**Figure 3:**
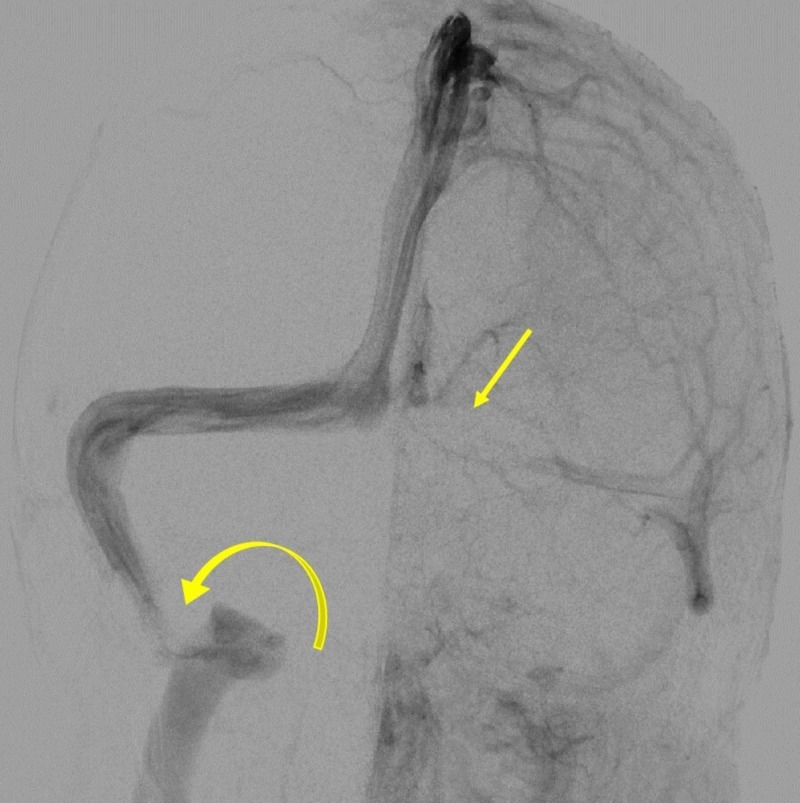
Diagnostic cerebral angiogram showing a filling defect within the right sigmoid sinus (curved yellow arrow) due to the mass lesion, and a hypoplastic left transverse sinus (straight yellow arrow).

The patient also underwent lumbar puncture with fluoroscopic guidance with an opening pressure of 45 mmHg. Diagnostic cerebral angiogram and venous manometry were also performed as follows: A single wall needle was utilized to access the right common femoral artery and using the Seldinger technique a 5-French sheath was placed and connected to continuous flush. Cerebral arteriography was performed with a 5-French catheter. Subsequently the right common femoral vein was accessed with a 5-French vascular access sheath which was connected to a continuous flush. Using a guidewire, a chaperone 6-French catheter was navigated to the level of the right internal jugular vein. Subsequently through the chaperone a Prowler catheter (Codman Neurovascular, New Brunswick, New Jersey, USA) was advanced over an Avigo guidewire (Medtronic, Minneapolis, Minnesota, USA) into the right superior sagittal sinus, right transverse sinus, right sigmoid sinus and right internal jugular vein with pressure measurements at each of those locations. Catheter and sheath were removed at the end of the procedure from both the right common femoral artery and vein. Findings included a filling defect within the right sigmoid sinus due to the mass lesion (Figure 3).

**Figure 4:**
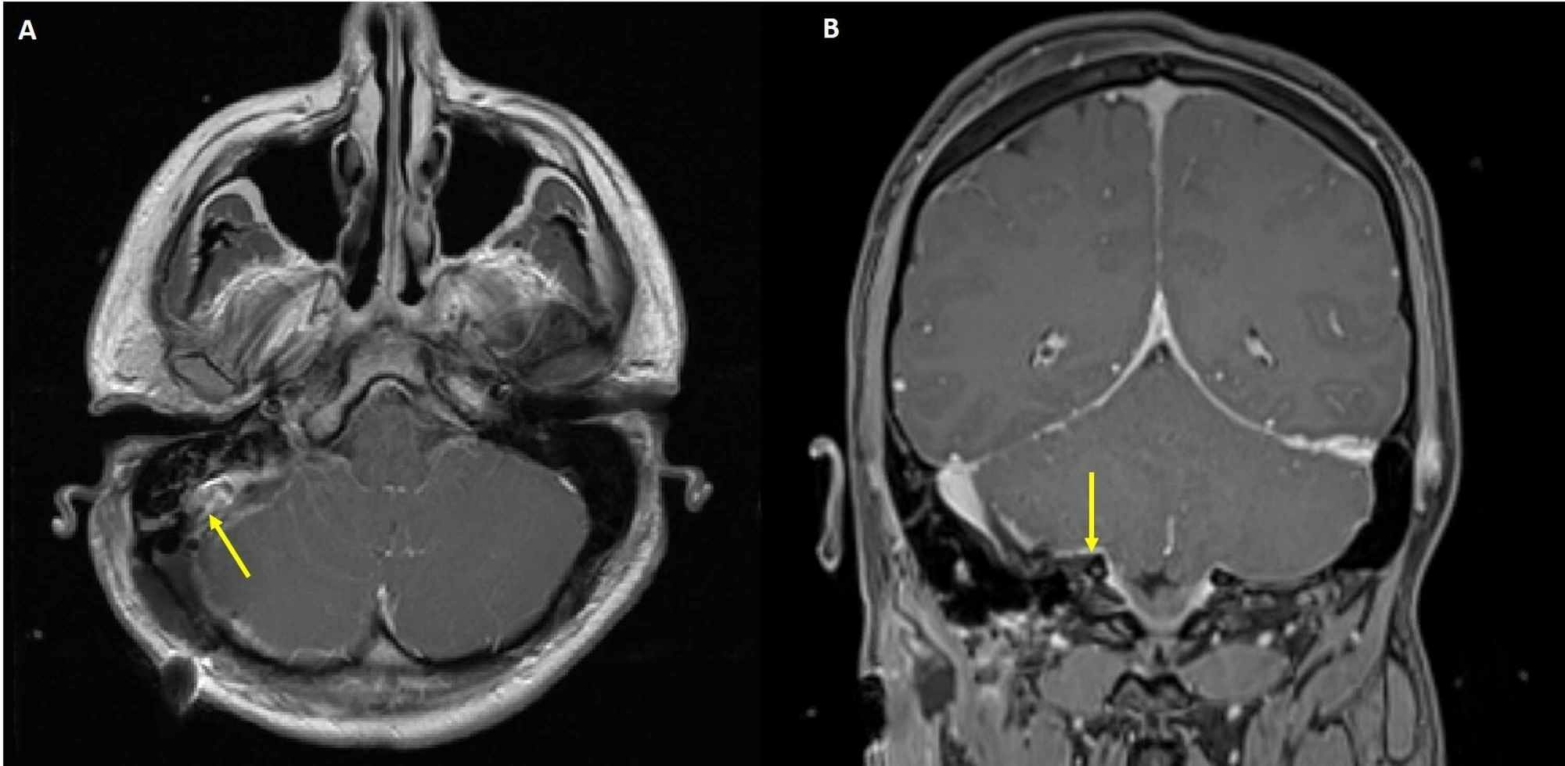
Post-operative magnetic resonance imaging (MRI) brain with gadolinium contrast (A) Axial and (B) coronal views showing partial resection of tumor at the right sigmoid sinus region (solid yellow arrows)

There was no delay in transit of contrast from the right straight sinus/ torcula to the right internal jugular vein. Manometry showed a central venous pressure of 36 mmHg (normal: 2-6 mmHg) within the right transverse sinus and 10 mmHg (normal: 2-6 mm Hg) within the right internal jugular vein (a 26 mm Hg pressure gradient across the mass on the right sigmoid sinus). Based on the patient’s clinical signs of increased intracranial pressure and the pressure gradient of 26 mm Hg (normal: <8 mm Hg) across the mass on the right sigmoid sinus, the decision was made to perform surgical debulking of the tumor followed by postoperative radiosurgery. Of note cerebral venous pressure gradient has been defined as ≥ 8 mmHg on venographic manometry [4]. An external ventricular drain was placed to drain cerebrospinal fluid for brain relaxation during surgery. A right-sided stereotactic retrosigmoid craniotomy was then performed, with the patient in the three-quarter prone position. Frozen section histology was consistent with a meningioma. Simpson grade IV resection of the tumor was carried out as this was considered safest in order to prevent injury to the dominant sigmoid sinus. Postoperative MRI brain with gadolinium contrast showed the residual tumor (Figure 4).

The final pathology of the tumor was reported as WHO grade I meningioma. Given the high rate of meningioma re-growth after Simpson grade IV resection, we decided to treat the residual tumor with stereotactic radiosurgery. The patient underwent Gamma Knife Radiosurgery to the residual meningioma at six months postoperatively, consisting of 12.5 Gy to the 50% isodose line with a total number of four isocenters. At immediate follow up, patient’s symptoms and papilledema had resolved. He remains asymptomatic at his 18-month follow-up.

## Discussion

Posterior fossa meningiomas constitute 7-10% of all meningiomas, with 10% involving the transverse or sigmoid sinuses [1]. Majority of patients with meningiomas are asymptomatic. However, symptomatic patients often present with symptoms of mass effect such as headache as well as symptoms peculiar to location. Invasion of the venous sinuses by a meningioma can cause obstruction of venous drainage leading to increase in intracranial pressure and papilledema. In this report, we described the management of a 17-year-old male who presented with intracranial hypertension caused by a meningioma at the right dominant sigmoid sinus. To the best of our knowledge the cerebral venous pressure gradient has not been recorded in any such cases.

Intracranial hypertension is defined by a sustained increase in the intracranial pressure of more than 20 cm H2O [6-7]. Intracranial hypertension caused indirectly by a mass compressing part of the intracranial dural sinuses, resulting in obstruction of venous drainage, has been described [2-3]. Intracranial hypertension due to a mass compressing the dural sinuses should be distinguished from idiopathic intracranial hypertension which requires an increase in the intracranial pressure of more than 20 cm H2O, in the setting of normal ventricular size, absence of an intracranial mass and absence of neurological deficits [8]. The mechanism by which compression of the dural sinuses result in increased intracranial pressure is explained by the modified Kellie-Monroe doctrine, which states that the sum of intracranial volumes is constant, and that an increase in any component must be offset by an equal decrease in another component [9]. Cerebrospinal fluid is absorbed at the arachnoid granulations based on a pressure gradient [10]. In dural sinus compression, there is increase in venous sinus pressure proximal to the site of obstruction, resulting in reversal of the arachnoid granulation-venous sinus pressure gradient and impairment of cerebrospinal fluid absorption [1]. Venous congestion with impairment of the cerebrospinal fluid absorption results in intracranial hypertension.

The diagnosis of intracranial hypertension is based on signs and symptoms of increased intracranial pressure, brain imaging and objective demonstration of increased cerebrospinal fluid pressure. Our patient presented with classic clinical findings of chronic headache and papilledema. Papilledema was demonstrated on fundoscopy and also on brain imaging. Passi et al. described classic MRI findings in papilledema [5]. Our patient demonstrated all these findings, which included flattening of the posterior sclera, enhancement of the prelaminar optic nerve, distension of the peri-optic subarachnoid space, intraocular protrusion of the prelaminar optic nerve and vertical tortuosity of the orbital optic nerve. Lumbar puncture confirmed elevation of cerebrospinal fluid pressure. Venous manometry is a very useful quantitative adjunct in confirming venous obstruction and in planning of treatment options such as stenting [11]. It is especially important in cases where symptoms of intracranial hypertension are less obvious. Our patient underwent venography and manometry and was found to have a pressure gradient of 26 mmHg across the mass on the right sigmoid sinus.

One of the goals of treatment for intracranial hypertension caused by a meningioma compressing the dural venous sinus is to relieve the obstruction. Options for achieving this include surgical resection, stereotactic radiosurgery, stenting or a combination of these modalities. The criteria for venous stenting include central cerebral venous pressure of ≥22mm Hg and a pressure gradient of at least 8 mmHg. Venous stenting for intracranial hypertension is minimally invasive and is especially useful when the patient is considered high risk for open cranial surgery [11-13]. Even though our patient met the criteria for venous stenting, stenting was not offered because of the potential for re-stenosis from continued tumor growth and intimal hyperplasia. Another disadvantage of the option of stenting, especially in this pediatric patient, would be the need for long-term antiplatelet therapy to prevent stent thrombosis. Our patient was offered surgical debulking of the meningioma (Simpson grade IV) followed by stereotactic radiosurgery to the residual tumor.

Mazur et al. described the nuances of surgical management of meningiomas involving the transverse or sigmoid sinus [3]. During surgical planning it is important to determine the degree of intravascular tumor invasion, the degree of sinus occlusion and the anatomy of venous outflow. This is achieved with preoperative imaging modalities including magnetic resonance angiography, computed tomography (CT) angiography and cerebral angiography. Cerebral angiography is particularly important in delineating the venous anatomy in the case of a unilateral dominant transverse-sigmoid sinus involvement [14]. Cerebral angiography revealed that our patient had a filling defect within the right sigmoid sinus due to the mass lesion. There was no delay in transit of contrast from the right straight sinus/torcula to the right internal jugular vein. There was also no arterial supply from the intracranial circulation, but there was a small tortuous feeding vessel from the ascending pharyngeal artery. The left transverse sinus was hypoplastic. The occurrence of the tumor around a dominant right sigmoid sinus with partial occlusion partly informed our conservative approach to tumor resection. Care was taken during surgery to preserve bridging veins so as to avoid postoperative edema and venous infarction. The tumor was extravascularly debulked without opening the sinus. Inadvertent opening of the venous sinus is associated with increased risk of air-embolism. In this case, injuring the dominant sinus would have been riskier given that even compression was resulting in intracranial hypertension.

Stereotactic radiosurgery for meningiomas at the transverse or sigmoid sinus is rarely reported, and this report is one of the few reported. Nowak et al. reported the surgical management of four jugular foramen meningiomas by transcondylar approach with sigmoid sinus ligation. One of the patients underwent stereotactic radiosurgery postoperatively for residual tumor [15]. Nicolato et al. also reported on their experience of gamma knife radiosurgery for 62 posterior fossa meningiomas but none of the meningiomas were at the sigmoid sinus [16]. Possible complications to radiosurgery could include edema from irradiation of peritumoral veins. However, excellent meningioma control has been reported with stereotactic radiosurgery [17]. Our patient had resolution of symptoms of intracranial hypertension including the papilledema and there has been no tumor regrowth during the admittedly short follow-up of 18 months.

## Conclusions

Sigmoid sinus meningioma can cause intracranial hypertension. Conservative surgical resection followed by stereotactic radiosurgery offers immediate relief of symptoms and excellent tumor control while avoiding the morbidity of a more radical surgical approach. In addition, using venographic manometry provides confirmation of the pathophysiology of intracranial hypertension in this patient.
